# Optimization and Characterization of Paper-Made Surface Enhanced Raman Scattering (SERS) Substrates with Au and Ag NPs for Quantitative Analysis

**DOI:** 10.3390/ma10121365

**Published:** 2017-11-28

**Authors:** Silvia Dalla Marta, Chiara Novara, Fabrizio Giorgis, Alois Bonifacio, Valter Sergo

**Affiliations:** 1 Department of Engineering and Architecture, University of Trieste, I-34127 Trieste, Italy; silviadallamarta@hotmail.it (S.D.M.); sergo@units.it (V.S.); 2 Department of Applied Science and Technology, Politecnico of Torino, I-10129 Torino, Italy; chiara.novara@polito.it (C.N.); fabrizio.giorgis@polito.it (F.G.)

**Keywords:** paper, nanoparticles, SERS substrates

## Abstract

In this work, we present a systematic study on solid Surface Enhanced Raman Scattering (SERS) substrates consisting of Au and Ag nanoparticles (NPs) loaded on filter paper with the dip-coating method. The aim of this work is to explore how a series of parameters (e.g., concentration of colloidal solution, different porosity of filter paper, and the presence of an aggregating agent) affects the analytical performance of paper-based SERS substrates. All the substrates developed in this study have been analyzed with two non-resonant probe molecules, 4-mercaptobenzoic acid (4-MBA) and adenine, in terms of (i) inter-sample repeatability, (ii) intra-sample repeatability, (iii) sensitivity, and (iv) overall SERS performance in terms of analyte quantification. Moreover, the issue of how to evaluate the repeatability for a solid SERS substrate is carefully discussed.

## 1. Introduction

Lately, the use of Surface-Enhanced Raman Spectroscopy (SERS) with solid substrates for sensing has been growing considerably [1,2,3,4,5,6,7,8]. The power of SERS relies on retaining the advantages of normal Raman, such as high specificity, coupled with the possibility to work in an aqueous environment while overcoming the limits due to a low sensitivity. This can be achieved by exploiting the plasmonic properties of nanostructured metal surfaces. Au and Ag nanoparticles (NPs) are examples of metal nanostructures that are able to enhance the Raman signal by several orders of magnitude for energy excitation close to their plasmon resonances [9,10,11]. The efficiency and rapidity of SERS measurements, its ease of use and the availability of portable instruments are some of the most distinctive advantages of this technique. However, in spite of the many pros of SERS, there are still challenges to be faced on the way to its widespread and routine use as a reliable analytical technique. In this sense, one of the main well-recognized challenges for the SERS community is the development of inexpensive, repeatable, easy to handle, and durable solid SERS substrates [1,2,12,13,14].

Paper-based SERS substrates, in which metallic NPs are deposited or grown on a network of cellulose fibers, ensure many advantages such as low-cost, flexibility, high surface to volume ratio, and high sensitivity. At the same time, they do not require complex fabrication methodologies such as physical vapor deposition (PVD), electron beam lithography (EBL), focused ion beam (FIB) techniques, or electrochemical deposition or roughening [6,15]. In contrast to other solid supports for the deposition or in-situ synthesis of metallic NPs, such as homogeneous glass or silicon, the flexibility and the porous structure of the paper allows a broader range of analytical applications: for instance, it can be used as a swab [16] to analyze trace analytes on surfaces, or in a paper analytical device (PAD) [17,18,19], providing the detection of a target analyte present in a complex mixture [20]. Analytes loaded into the paper device by swabbing or dipping can be concentrated in a small region by exploiting the lateral flow concentration concept [21]. Moreover, the three-dimensional structure of the paper allows both high specific surface and inter-particle plasmon coupling, furtherly enhancing the SERS signal [22]. 

With the aim of providing substrates with such characteristics, in this work, we present paper-based substrates developed using dip-coating procedures [16,22,23], i.e., by depositing already-formed Au and Ag NPs on paper upon its prolonged immersion in a metal colloid. Moreover, in contrast with the in situ synthesis [24,25], an ex-situ synthesis allows a better control on the size, the shape and the chemical characteristics of the NPs surface [22,26,27,28]. The preparation method here described has been optimized not only taking into account the enhancing capability but also focusing on repeatability. In fact, for quantitative analysis applications, a low repeatability is one of the main issues hindering a widespread use of solid SERS substrates developed with a bottom-up self-assembling approach. Despite the promising results obtained so far on these type of substrates [26,27,28,29,30], there is a general lack of information about how experimental conditions affect their repeatability.

The very task of evaluating the repeatability of a solid SERS substrate is, in itself, nontrivial. Different types or levels of repeatability are involved, depending on what set of measurements have to be considered (intra- or inter-substrate). It is worth to emphasize that standard protocols backed by adequate statistics are still lacking for solid SERS substrates. In some cases, authors use the relative standard deviation (RSD, also known as coefficient of variation, CV) of the intensity or the area of a SERS band, reporting only the intra- or the inter-sample repeatability [28,31,32]. In few works, the RSD values for both intra- and inter-sample repeatability are reported [24,33,34], but in most of the available literature, the repeatability is not discussed at all [14,22,25,35,36]. In this work, repeatability has been assessed by collecting Raman maps for several replicas of substrates prepared with a specific set of parameters, and then calculating the mean as well as the RSD values from intensities integrated over one specific band (see Methods for details). 

Specifically, in this work, we explore how parameters such as paper porosity, NPs concentration, aggregating agents and excitation wavelength affect the repeatability of paper-based SERS substrates obtained from dip-coating procedures. Finally, we present in detail substrates optimized in terms of repeatability and sensitivity, aiming to build a platform for quantitative analysis. Moreover, for the first time, we present paper substrates decorated by the well-known and widely used Ag citrate-reduced NPs using a dip-coating method.

## 2. Materials and Methods

### 2.1. Materials and Reagents

All chemicals, solvents, and the filter paper with 20 µm of pore size (qualitative filter paper, grade 4 Whatman) were purchased from Sigma-Aldrich (Gallarate, Italy) and used as received. Filter paper with average pore size of 2 µm (qualitative filter paper, 410) were purchased from VWR international (Milan, Italy). Phosphate buffered saline solution (PBS) (pH 7.4) was prepared by dissolving one PBS tablet (Sigma-Aldrich) in Milli-Q water (200 mL). All glassware used for Au and Ag NPs preparation was carefully cleaned with aqua regia and thoroughly rinsed with Milli-Q water. For all cleaning procedures and preparation of solutions, Milli-Q water was used.

### 2.2. SERS Substrates Preparation and Characterization

Two types of metal NPs were used as nanostructured substrates: citrate-reduced Au and Ag NPs. Au NPs were synthesized according to a protocol described by Turkevich et al. [37].

Briefly, 10.6 mg of NaAuCl_4_ were dissolved in 25 mL of Milli-Q water and heated to boiling. 750 μL of 1% sodium citrate were then rapidly added to this solution under vigorous magnetic stirring. The solution was kept boiling under stirring for 20 min.

The citrate-reduced Ag NPs were synthesized according to a protocol described by Lee-Meisel et al. [38]. Briefly, 45 mg of AgNO_3_ were dissolved in 250 mL of Milli-Q water and heated to boiling. 5 mL of a 1% sodium citrate tribasic solution were then added dropwise to the AgNO_3_ solution under vigorous magnetic stirring. The solution was kept boiling under stirring for 1 h. Both Au and Ag colloidal solutions were stored in dark at room temperature (RT) and were stable for several months [39]. 

To obtain concentrated colloids, the colloidal dispersions obtained with those syntheses were concentrated using a minispin Eppendorf centrifuge for 30 min at 6000 rpm (2415× *g*) and 10,000 rpm (6708× *g*) for Au and Ag NPs respectively. Thus, 9 parts of the supernatant liquid above the NPs pellets was removed in order to reach a solution concentrated 10 times in volume.

The colloids were characterized by UV-visible absorption spectroscopy after each preparation using a Lambda 20bio UV–Vis spectrometer (Perkin-Elmer, Milano, Italy). Au NPs feature a surface plasmon band at 540 ± 2 nm, while the Ag NPs at 410 ± 5 nm. All these values are consistent with the values previously reported in literature [40,41,42].

The colloids were also characterized by Transmission Electron Microscope (TEM, Philips EM 208, Amsterdam, The Netherlands). Both UV–Vis extinction spectra and TEM images of Au NPs and Ag NPs for different preparations can be found in the Figures S1 and S2 of the Supplementary Material. TEM micrographs show that the Au NPs have a spherical shape with a diameter of 50 nm, while the Ag NPs show a more varied shape distribution with respect to the Au NPs, with diameters ranging between 50 and 100 nm.

The solid SERS substrates were obtained with the so called “dip-coating” method, loading the NPs on the filter paper [16,22,23,28]. 

Scanning electron microscopy (SEM) secondary electrons contrast images of the SERS substrates were collected with 5 keV electrons using an in-lens detector of a Zeiss SUPRA 40 (Zeiss SMT, Oberkochen, Germany) field emission electron microscope (FE-SEM). A metallization step with a few nm thick Pt layer was needed before the FE-SEM analysis to improve electron discharging for the Ag NPs loaded substrates. 

In this work, we prepared eight different types of solid SERS substrates for both Au NPs and Ag NPs, in particular: (i) with not concentrated (i.e., as prepared) NPs and with a filter paper with an average pore size of 2 µm, (ii) with 10 times in volume concentrated NPs and with a filter paper with an average pore size of 2 µm, (iii) with not concentrated NPs and with a filter paper with an average pore size of 20 µm, and (iv) with 10 times in volume NPs with a filter paper with an average pore size of 20 µm. All these types of substrates were also prepared in the presence of 20 mM sodium citrate as aggregating agent. The choice of sodium citrate as aggregating agent is due to two different reasons: (i) citrate ions are already adsorbed onto the NPs surfaces from the synthesis reaction, and (ii) citrate adsorbs onto the NPs surfaces through relatively weak bonds, allowing the exchange with the target molecule and, hence, allowing its SERS detection. 

For the substrates prepared with the aggregating agent, a piece of 1 cm^2^ of paper with 2 or 20 µm of average pore size was placed on the bottom of a cylindrical glass vial (total capacity of 10 mL) containing 3 mL of Au or Ag colloidal solution (concentrated or not); then the sodium citrate was added up to a final concentration of 20 mM. The presence of the citrate leads to a color change of the colloidal solution from red to grey for Au NPs and from grey to dark grey for Ag NPs, indicating the NPs aggregation. The vials containing the colloid and the filter paper were then stocked in dark at room temperature for one week (“incubation”). During the incubation, the NPs deposited on paper on the vial bottom. After incubation, the colorless, transparent supernatant solution, devoid of nanoparticles, was removed with a plastic syringe, taking extra care not to touch or move the paper substrate. For the substrates developed without the aggregating agent, the same protocol above was applied, but without the addition of sodium citrate to the colloidal solution. The substrates were then left in the vials, dried for a few hours in air at room temperature and then stocked in Milli-Q water until used. We observed that the drying step makes the substrate more stable, so that once they are re-immersed again in aqueous solutions the NPs do not detach from the paper support.

### 2.3. Analytes Preparation for SERS Measurements

4-Mercaptobenzoic acid (4-MBA) and adenine were chosen as probe analytes for three reasons. First, the molecules are bound to the metal surfaces through different functional groups (through the thiol for 4-MBA [43] and through the purine nitrogen for adenine [44,45]). This allows a characterization and a sensitivity study of the substrates for both kind of chemical interactions. Second, both analytes are non-resonant for the applied excitation wavelengths. This allows a SERS characterization of the substrates, which does not depend on the nature of the analyte but only from the characteristic of the plasmonic structure, thus giving a better indication about their repeatability and sensitivity. Third, both analytes feature SERS spectra with an isolated, narrow and intense band (740 cm^−1^ for adenine and 1583 cm^−1^ for 4-MBA) generated by one vibrational normal mode (the ring breathing). This allows the analysis of the band areas without any interference from adjacent or convoluting bands, ensuring a better reliability of the experimental results. 

Mother solutions 1 mM of 4-MBA and adenine were prepared dissolving the analytes in MeOH and 1 M aqueous NaOH, respectively. For the measurements of repeatability, the mother solutions were diluted to a final analyte concentration of 10 μM in PBS in order to reach a pH of 7.4. The 10 μM concentration was chosen to test the repeatability performance of the substrates used for quantitative analysis. For the calibration curves, analyte solutions were prepared in a concentration range of 0.1–40 μM in PBS. 

For all experiments, each substrate (as prepared, 1 cm^2^) was cut in four pieces of 5 mm × 5 mm, which were incubated by immersion in the analyte solution for 5 min. After the incubation, the samples were gently rinsed with Milli-Q water in order to remove the loosely bound molecules and the excess molecules not directly adsorbed onto the NPs surface, and then dried for 15 min at RT in air before SERS analysis. 

### 2.4. Instrumentation, SERS Spectra, and Images Acquisition

SERS spectra were recorded with a Renishaw inVia Raman microscope (Renishaw plc, Wotton-under-Edge, UK) equipped with a microscope Leica DMLM with a 10× objective (N.A. 0.25). Excitation was obtained with a 785 nm diode laser (Toptica AG Photonics, Graefeling, Germany), with an output power of 500 mW (2 mW at the sample) and a 514.5 nm Modulaser Ar Stellar-Pro ion laser (Laser Physics UK Ltd, Cheshire, UK), with an output power of 50 mW (5 mW at the sample). The spectrograph was equipped with a 1200 lines/mm (for 785 nm excitation) or with an 1800 lines/mm (for 514.5 nm excitation) grating and a cooled charge coupled device (CCD) detector. The frequency calibration for all gratings was done using the emission lines of a Ne lamp. All data were collected using the WiRE software (Version 3.2, Renishaw plc, Wotton-under-Edge, UK).

To facilitate handling, before each measurement, each 5 mm × 5 mm paper substrate was adhered to a standard glass microscope slide (25 mm × 75 mm), which was then immobilized onto the microscope stage. 

For the repeatability study, for each substrate a set of 64 SERS spectra was acquired from a square grid of 8 × 8 points, with a step between adjacent points of 50 µm, yielding a small map. All maps were acquired using the “static” acquisition mode of the WiRE software, centered on the SERS band of interest. In all maps, spectra were recorded using a single scan (5 s of exposure time for each spectrum). SERS spectra for the calibration curves were recorded using the “extended” acquisition mode of WiRE software, over a wavenumber range from 600 to 1500 cm^−1^ for adenine and from 800 to 1700 cm^−1^ for 4-MBA; all spectra were recorded using one accumulation (10 s exposure) at 7 random locations on the sample. 

To avoid photodegradation, the laser power density at the sample was decreased for both lasers by increasing the diameter of the laser spot using the “defocusing” option (at a value of 100%) of the InVia Raman microscope (Renishaw plc, Wotton-under-Edge, UK). 

### 2.5. Data Preprocessing, Analysis, and Plotting

Spectra preprocessing (i.e., vector normalization and first polynomial baseline correction), analysis (i.e., calculation of mean and standard deviation for band areas), and plotting (i.e., SERS spectra, Tukey’s plots and calibration curves) were performed using the hyperSpec package [46], the baseline package [47], and chemCal package [48] for R [49]. 

In order to obtain a reliable comparison of the band areas obtained with different laser sources, all the spectra were normalized with respect to the value of the intensity of the Si band (520 cm^−1^) recorded with the laser used. Limits of Detection (LOD) and of Quantification (LOQ) were calculated with the chemCal package [48], according to the standard IUPAC definitions of LOQ and LOD as found in the IUPAC Gold Book (goldbook.iupac.org).

Intra- and inter-sample Relative Standard Deviations (RSD) of the 4-MBA band area (1550~1615 cm^−1^), and of the adenine band area (700~770 cm^−1^) were calculated from SERS maps for all kind of substrates under investigation. The inter-sample repeatability was obtained from the integrated intensity averages calculated for each map of the same kind of substrate.

## 3. Results and Discussion

### 3.1. SEM Characterization of Substrates 

**Figure 1:**
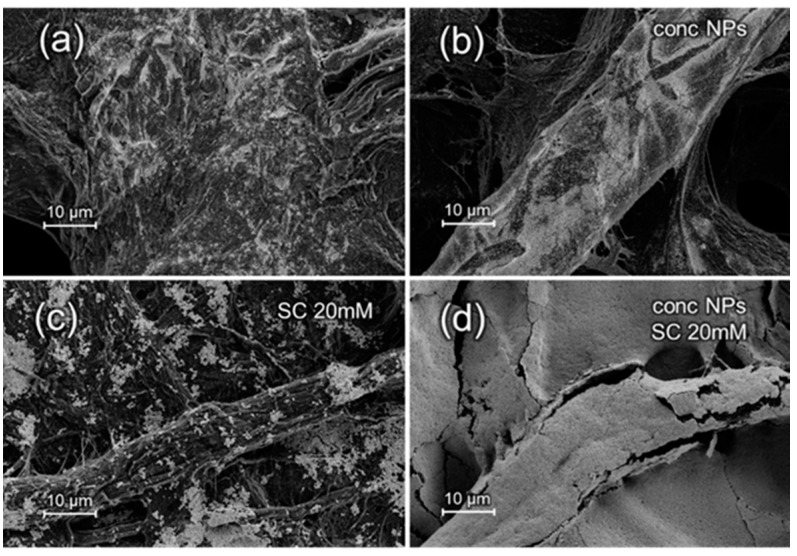
Field emission electron microscope (FE-SEM) images with the same magnification (5 k) of Ag nanoparticles (NPs), as prepared (**a**,**c**) or 10× concentrated (“conc NPs”) (**b**,**d**), loaded on filter paper (average pore size: 2 μm) in absence (**a**,**b**) or presence (**c**,**d**) of 20 mM sodium citrate (SC).

**Figure 2:**
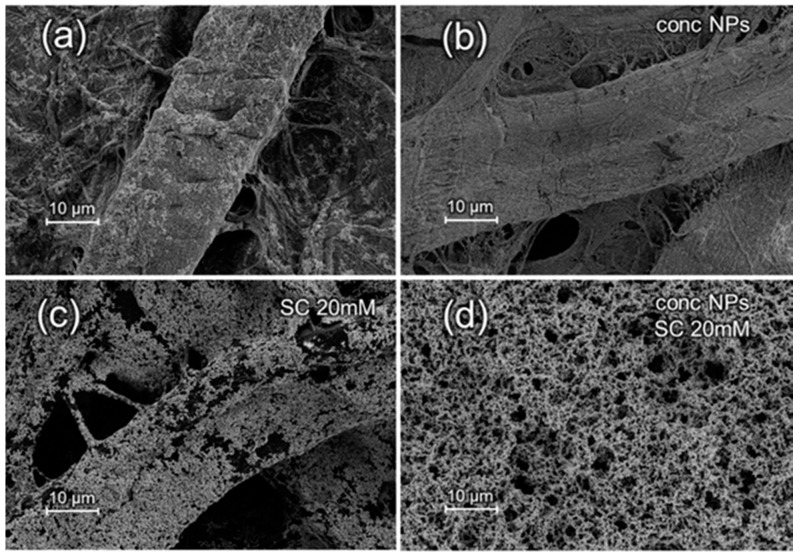
FE-SEM images with the same magnification (5 k) of Au NPs, as prepared (**a**,**c**) or 10× concentrated (“conc NPs”) (**b**,**d**), loaded on filter paper (average pore size: 2 μm) in absence (**a**,**b**) or presence (**c**,**d**) of 20 mM sodium citrate (SC).

Ag and Au SERS substrates were characterized with FE-SEM, and the obtained images are reported in Figure 1 and Figure 2, respectively. An FE-SEM comparison of Ag and Au substrates images at higher magnification is reported in Figures S3 and S4 of the Supplementary Material. 

For both Au and Ag substrates, it is possible to observe the effect of NPs concentration as well as of the presence of an aggregating agent on the final surface morphology. The density of NPs adsorbed onto the cellulose fibers increases with NPs concentration, as observed for Au NPs in the paper presented by Ngo et al. [22], as well as in presence of sodium citrate, consistently with the results presented in the work of Mehn et al. [27]. Hasi et al., with the same procedure, presented substrates loaded with citrate-reduced Ag NPs [26], increasing the ionic strength with the use of chloride ions to overcome the electrostatic repulsion between the negatively charged carboxyl groups of the cellulose and the citrate-coated NPs. However, SEM images in Figure 1 clearly demonstrate the feasibility to load citrated-reduced Ag NPs on filter paper with the dip-coating method without the need of chlorides. The uniform and high-density adsorption of the Ag NPs does not seem to be hampered by the electrostatic repulsion between the negatively charged NPs and the carboxyl groups of the cellulose. For both Au and Ag NPs, the synergic effect of NPs concentration and aggregating agent leads to a dense layer of deposited NPs which masks, especially in the case of Au, the fiber-like structure of the cellulose substrate. The NPs layer is rather uniform, i.e., it covers most of the cellulose surface, at larger scales (i.e., hundreds of micrometers), especially for Au NPs and when concentrated NPs are used (Figures S5 and S6 in Supplementary Material).

### 3.2. Effect of Paper Porosity, Aggregating Agents and Nanoparticles Concentration on Substrates Repeatability 

**Figure 3:**
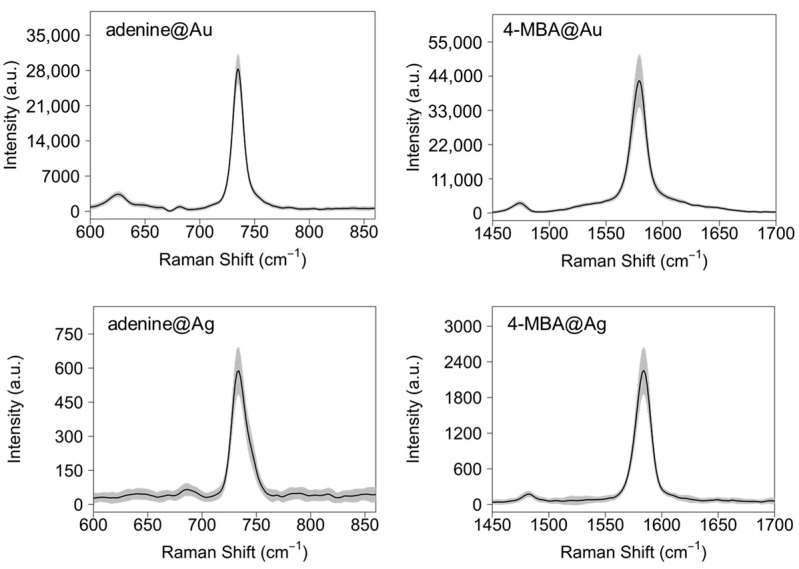
Mean SERS spectra of the most intense bands of adenine and 4-mercaptobenzoic acid (4-MBA) acquired on Au and Ag substrates, calculated from maps consisting of 64 (i.e., 8 × 8) spectra acquired at 785 nm for the Au substrates and at 514.5 nm for the Ag substrates. Shaded areas represent ±1 intensity standard deviation

The substrates obtained in different conditions of colloid concentration, paper porosity and presence of sodium citrate were investigated in terms intra- and inter-samples repeatability of the selected band areas. Mapping of 4 replicas of each type of substrate was performed after samples incubation in 4-MBA or adenine PBS solutions (see Methods). For all Ag substrates, maps were collected with the 514.5 nm and 785 nm excitation; for Au substrates, since neither 4-MBA nor adenine spectral signature could be detected at 514.5 nm, all the data were collected with 785 nm laser. In Figure 3, we report, as examples, the average SERS spectra of the most intense bands of adenine and 4-MBA of Au and Ag substrates obtained from as-prepared colloids deposited on a filter paper with an average pore size of 2 µm, in presence of sodium citrate as aggregating agent. All average SERS spectra acquired from all kinds of Au and Ag substrates, with different excitation wavelengths, are reported in Figures S7–S10 in the Supplementary Material. The SERS bands of adenine and 4-MBA in Figure 3 are consistent with those already reported in literature for these two substances [43,45]. 

**Figure 4:**
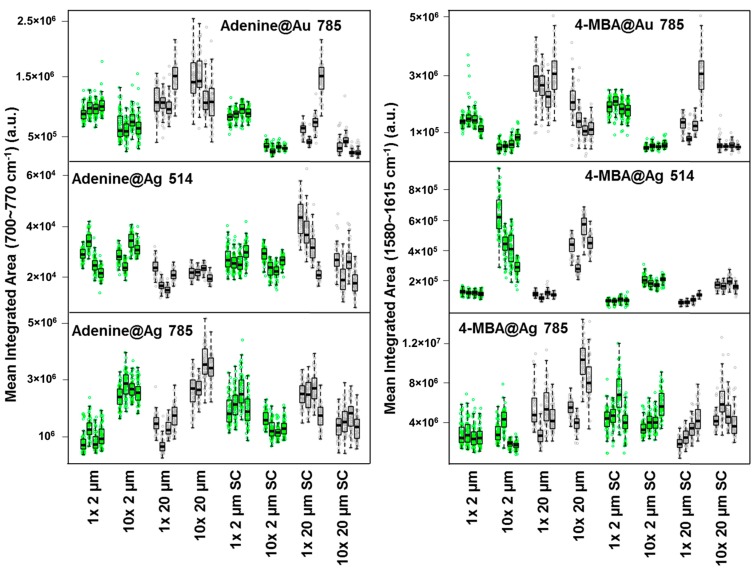
Comparison of the distributions of SERS intensities integrated over the area of the adenine and 4-MBA band. Each box represents the complete distribution of integrated intensities, calculated over a SERS map. Boxes are grouped according to analyte, metal and excitation wavelength, with four replicas (i.e., boxes) for each set of conditions (NPs conc: 1×, 10×; paper average pore size: 2, 20 µm, presence of sodium citrate: SC). For the distributions, the intra- and inter-sample Relative Standard Deviation (RSD%) values are reported in Table 1, Table 2 and Table 3. Different colors correspond to different average paper porosity (green for 2 µm, grey for 20 µm). Along the median value, the first and the third quartiles of the data distributions are indicated, as bottom and top of the boxes.

From these maps, intensity data were derived by integrating the intensity over the band area. So far, in most of the works on paper-based SERS substrates, the inter- and intra-sample repeatability is only characterized using numerical RSD values, losing the information about the characteristics of the intensity distributions [24,28,32,34]. Other authors graphically represent the data without discussing the intensity distributions obtained [26,27,31,33]. Moreover, in most cases, the repeatability analysis of the samples is not complete since only the intra- or the inter-sample repeatability is reported [26,27,31,32], while in our opinion, both are key-parameters. Finally, spectra are often acquired from few spots on each substrate, possibly leading to a statistically inaccurate intra-sample repeatability [24,26,28,31,32,34]. To increase the information content on SERS data from these substrates, we chose to present the results from the acquired maps with Tukey’s plots. This kind of box plot allows to visually estimate the distribution characteristics of the experimental data, representing the distribution of the integrated areas for each spectrum acquired in a map. The results were compared in the Tukey’s box plot (1 box = 1 map) of Figure 4 in terms of intra- and inter-sample intensity repeatability for each kind of substrate, excitation wavelength and probe molecule. 

Information on both intra- and inter-sample repeatability can be inferred from Figure 4. In particular, intra-substrate repeatability is related to the interquartile range (i.e., the “height”) of the single boxplot, while inter-substrate repeatability can be appreciated by comparing the 4 boxplots representing the four replicas. The direct comparison between different boxes immediately provides an estimate of different aspects of intra- and inter-substrate repeatability. However, to allow a better comparison with literature data (where in most cases the data variability is expressed using relative standard deviation) we also reported the RSD% values for each measurement (i.e., for each map) in Table 1, Table 2 and Table 3. The RSD% was calculated by dividing the standard deviation (S) by the average integrated intensity (M) obtained from the maps. In particular, for the intra-sample repeatability values, S and M were the standard deviation and the mean integrated area calculated from the 64 spectra acquired for each map. For the inter-sample repeatability, the RSD values were evaluated among the four replicas sharing the same experimental conditions, and S and M were calculated over the mean values of the distributions of the four replicas.

In terms of signal enhancement, intensity is higher when exciting Ag NPs with 785 nm with respect to the excitation at 514.5 nm. No other clear trend on intensity can be observed upon varying NPs concentration, paper porosity or presence of aggregating agent. In Figure 4, the variation of the mean integrated area among different groups is most marked for 4-MBA investigated on Au at 785 nm and on Ag at 514 nm. Interestingly, the choice of laser excitation wavelength and the type of metal had a significant impact on the overall intensity, dominating over other parameters such as NPs concentration, paper porosity and presence of citrate. For instance, substrates prepared from concentrated Ag NPs, upon 514 nm excitation, display a higher intensity than those prepared from non-concentrated NPs, whereas for the Au substrates investigated with a 785 nm excitation the opposite is true. As far as repeatability is concerned, the only clear pattern is that Ag substrates excited with 514.5 nm show a better repeatability (see Table 2 and Table 3).

From the Tukey’s plots and the RSD values reported in Table 1, Table 2 and Table 3, it is also possible to observe that, as a general trend, the substrates developed with a less porous paper present better intra- and inter-sample repeatability. These results apparently disagree with those obtained by Cheng et al. [24]. In that work in fact, the authors observed a negligible variation between SERS signals acquired on filter paper with different pore sizes. This general behavior of the paper-based substrates could be explained with their intrinsic three-dimensional structure. A smalleraverage pore size of the paper implies a denser structure of the fibers on which the NPs are adsorbed. Thus, within the area scanned by the spot of the laser, the surface is more homogenous and less irregular with respect to the paper with 20 µm of porosity, also in terms of SERS hot spot distributions. The consequence is a minor variation of the laser focus, hence a minor variation of the SERS signal among spectra acquired within a map, as well as among different maps. 

On the other hand, NPs concentration and presence of citrate do not show any general trend, and their effect on the intensity and repeatability vary depending on the specific combination of metal and excitation wavelength. In some cases, also the comparison with the available literature is somewhat problematic. For instance, for similar paper-made substrates Ngo et al. observed that the signal increases with the Au colloid concentration [22], while we observe an opposite trend, with the exception of the combination with the filter paper with 20 μm of average porosity (which follow the behavior reported by Ngo et al.).

The presence of the citrate as aggregating agent on the RSD is not as relevant as one would expect by observing the SEM images reported in Figure 1 and Figure 2. Those images in fact show a more extensive coverage of the paper in the presence of citrate. Therefore, we expected a general improvement on the repeatability due to the better surface homogeneity. Instead, the intra- and inter-sample repeatability values do not clearly correlate with the presence of the aggregating agent, which is somewhat in disagreement with what reported by Mehn et al. for Au NPs deposited on paper [27].

From Figure 4 and Table 1, Table 2 and Table 3, it is possible to find which combination of parameters lead to most performing Au and Ag substrates in terms of intra- and inter-sample repeatability and SERS intensity. Independently from the kind of analyte used as probe molecule, the Au substrates featuring the best repeatability are those developed using (i) paper with an average pore size of 2 μm,(ii) in presence of citrate and (iii) with an as-prepared (i.e., non-concentrated) colloidal dispersion. Such kind of substrates feature an intra-sample RSD between 9% and 16% and an inter-sample RSD of 6%. The most repeatable Ag substrates were obtained with the same conditions but using a concentrated colloidal solution. Such substrates, upon 514.5 nm excitation, feature an intra-sample repeatability between 8% and 14% and an inter-sample repeatability between 9% and 12%. For most purposes, such values allow to perform a quantitative SERS analysis. 

### 3.3. Estimation of the Raman Enhancement Factor (EF)

Since its difficult to determine the effective SERS active area of the substrates as well as the number of molecules adsorbed on the NPs, a practical EF have been determined by the following expression, as reported in literature [35,50]:

$$\mathrm{EF}=\left(\frac{I_{\mathrm{SERS}}}{I_{\mathrm{Raman}}}\right)\left(\frac{M_{\mathrm{Raman}}}{M_{\mathrm{SERS}}}\right)$$

where the intensity I is the average height of a selected band (the 1583 cm^−1^ band of 4-MBA or the 740 cm^−1^ band of adenine) in normal Raman and SERS spectra, and M is the molarity of the adenine and 4-MBA solutions. The EF were calculated for Au and Ag substrates developed with the optimized conditions (filter paper with an average pore size of 2 μm, with 20 mM of sodium citrate, with not concentrated Au NPs and 10× concentrated Ag NPs). We thus measured the Raman signal of 10 µM adenine and 4-MBA on the substrates, and the Raman signal of 0.3 M of adenine and 0.5 M of 4-MBA dried on the paper substrates without NPs.

It was not possible to determine an EF for Ag substrates with the 514.5 nm laser because of the fluorescence background produced by the cellulose, which totally covered the Raman signal of the analyte on the bare support. Therefore, EF were calculated only for 785 nm excitation. The EF determined in this way for both adenine and 4-MBA were found to be approximately 4 × 10^3^ for Au substrates, and 3 × 10^7^ for Ag substrates. Similar results for non-resonant analytes are reported in literature for paper loaded with spherical AuNPs [27], while for non-spherical Au NPs (i.e., nanorods) the EF reported are rather larger [16,28]. In summary, the Ag substrates presented in this work show an EF that is two orders of magnitude larger compared with that of the Ag substrates developed by Hasi et al. [26]. 

### 3.4. Adenine and 4-MBA Quantification 

**Figure 5:**
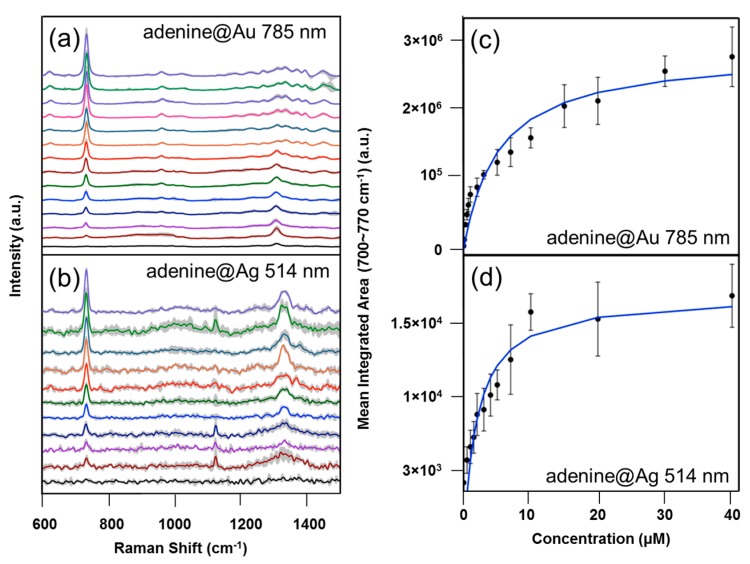
(**Left**) Average SERS spectra (±1 intensity standard deviation as grey shaded areas) at several concentrations of adenine on (**a**) Au substrates, at 785 nm and on (**b**) Ag substrates at 514.5 nm excitation. (**Right**) Adsorption curves of (**c**) adenine on Au and (**d**) on Ag, fitted with Langmuir isotherms (error bars represent ±1 standard deviation over spectra collected from seven different random locations on a substrate).

**Figure 6:**
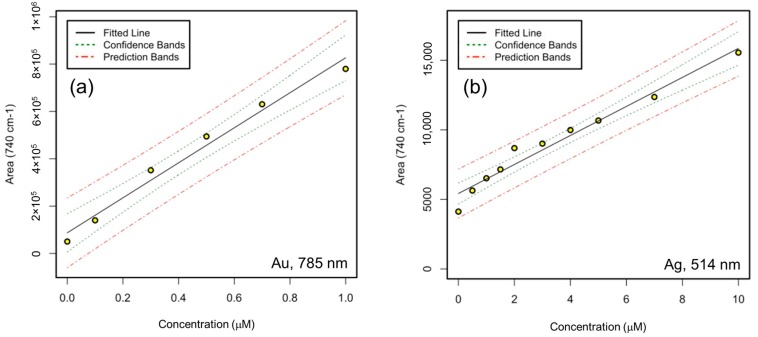
Calibration curves with 95% confidence (uncertainty in the curve estimation) and prediction (uncertainty about the new values on the curve) intervals for concentration ranges with a linear response, for adenine on Au substrates (**a**) at 785 nm and on Ag substrates (**b**) at 514.5 nm. Yellow-filled black represent the values of the integrated areas of the adenine band centered at 740 cm^−1^.

The use of these substrates for quantitative analysis was assessed by studying the adsorption curves of adenine, reported in Figure 5, and of 4-MBA, (reported in Figure S11 on the Supplementary Material). As for the EF calculation, we selected the parameters featuring the best repeatability for both Au and Ag substrates. The adsorption curves were obtained by plotting the mean integrated area over the 700~770 cm^−1^ band at different concentrations of analyte. The average SERS spectra of adenine on both Ag and Au substrates are reported in Figure 5a,b (data for 4-MBA are reported in Figure S11a,b in Supplementary Material). SERS spectra were obtained by incubating the substrates in aqueous solutions at different concentration of the analyte, from the lowest detectable concentration up to concentrations where the SERS intensity approaches saturation, due to the formation of a monolayer and the depletion of the surface sites available for adsorption. These two analytes work as model analytes for the application of these substrates to quantitative analysis, and univariate calibration curves can be constructed for specific concentration ranges where the response is linear (Figure 6 and Figure S4c,d). LOD and LOQ for adenine on Au substrates are approximately 0.3 and 0.5 µM, respectively, while for Ag substrates these values are one order of magnitude higher. For 4-MBA, LOD and LOQ on Au substrates are 2 and 5 µM, whereas on Ag substrates they are 1 and 2 µM, respectively. The zero concentration values in the calibration curves were calculated from the background spectra of the substrates without the analyte (see Figure S12 in Supplementary Material). The complete *R*^2^ values, the LODs and LOQs derived from the calibration curves are reported in Table S1 for the two analytes and the two substrates under investigation. These values reflect the different affinity of these two analytes for the two metals: for adenine it is higher for the Au than for the Ag substrate, whereas 4-MBA shows the opposite trend. 

## 4. Conclusions

In this work, a systematic study on paper-based Au and Ag solid SERS substrates, prepared with the dip-coating method, was performed by observing their behavior with two different non-resonant analytes. Experimental parameters, and in particular excitation wavelength and paper porosity, indeed affect the substrates performance in terms of overall efficiency (i.e., enhancement factors) and repeatability. On the other hand, the effect of parameters such as NPs concentration and presence of citrate vary depending on the specific combination of plasmonic metal and excitation wavelength, lacking a definite trend. The Au substrates featuring the highest repeatability were developed with a filter paper with an average pore size of 2 µm, using an as-prepared (i.e., not concentrated) colloidal dispersion and in presence of 20 mM of sodium citrate as aggregating agent. On the other hand, the most repeatable Ag substrates were developed using a pre-concentrated colloidal dispersion while keeping all the other parameters the same as for Au. Au substrates developed this way show an excellent repeatability, with an intra-sample RSD between 9% and 16% and inter-sample RSD of 6%. Ag substrates showed a slightly lower repeatability, but still with intra-sample RSDs below 20% and an inter-sample RSD of 7%. Repeatability, however, has to be traded at the expense of sensitivity, since Au substrate show a lower EF than Ag substrates. The reported results show that, by tuning experimental parameters, substrates prepared with a bottom-up approach using inexpensive materials and procedures, such as dip-coating of paper with metal colloids, can be effectively used for reliable quantitative SERS analyses.

## Figures and Tables

**Table 1:** Intra- and inter-sample Relative Standard Deviations (RSD) of the 4-MBA band area (1550~1615 cm^−1^) and of the adenine band area (700~770 cm^−1^) calculated from SERS maps for Au substrates excited at 785 nm.

Au Substrates—785 nm Excitation			RSD% Intra Sample (*Inter-Sample*)	
	Average Pore Size	NPs Concentration	w/o Citrate	with Citrate
**4-MBA**	2 µm	1×	10.8, 27.3, 14.1, 14.3 (*13.4*)	12, 13.7, 16.2, 15.6 (*6.2*)
		10×	27.9, 14.3, 26.9, 19.4 (*23.7*)	14.7, 16.0, 10.4, 17.9 (*7.3*)
	20 µm	1×	27.5, 19.8, 20.4, 23.7 (*14.4*)	17.9, 22.7, 18.2, 23.7 (*60.6*)
		10×	27.5, 32.0, 25.3, 26.6 (*34.1*)	32.0, 11.3, 21.1, 11.5 (*6.4*)
**Adenine**	2 µm	1×	13.4, 12.6, 12.5, 14.8 (*6.1*)	12.5, 9.3, 12.8, 9.8 (*6.0*)
		10×	34.3, 24.5, 19.4, 31.5 (*10.7*)	14.9, 21.6, 11.7, 9.8 (*11.7*)
	20 µm	1×	21.8, 17.0, 18.4, 18.5 (*19.3*)	13.8, 13.4, 17.3, 18.5 (*57.6*)
		10×	26.6, 24.2, 24.0, 31.5 (*17.0*)	35.3, 25.6, 18.9, 24.5 (*32.3*)

**Table 2:** Intra- and inter-sample Relative Standard Deviations (RSD) of the 4-MBA band area (1550~1615 cm^−1^), and of the adenine band area (700~770 cm^−1^), calculated from SERS maps for Ag substrates excited at 514 nm.

Ag Substrates—514 nm Excitation			RSD% Intra Sample (*Inter-Sample*)	
	Average Pore Size	NPs Concentration	w/o Citrate	with Citrate
**4-MBA**	2 µm	1×	11.0, 9.8, 12.1, 12.4 (*4.4*)	16.7, 15.9, 15.7, 18.3 (*6.6*)
		10×	21.0, 17.4, 24.0, 15.4 (*31.6*)	13.9, 11.6, 9.6, 9.4 (*9.1*)
	20 µm	1×	12.4, 11.6, 14.1, 9.5 (*12.1*)	17.8, 16.9, 16.8, 9.5 (*26.8*)
		10×	12.8, 12.1, 12.8, 11.3 (*25.6*)	12.8, 13.3, 15.5, 12.5 (*9.4*)
**Adenine**	2 µm	1×	8.4, 9.7, 12.6, 11.8 (*20.0*)	16.7, 13.7, 12.8, 13.4 (*7.4*)
		10×	9.7, 9.1, 9.8, 8.5 (*14.7*)	10.4, 13.0, 12.2, 8.4 (*12.5*)
	20 µm	1×	12.9, 12.3, 10.4, 10.7 (*21.8*)	15.5, 17.2, 17.0, 10.7 (*29.0*)
		10×	11.4, 8.2, 7.2, 8.8 (*7.6*)	19.0, 31.1, 21.4, 26.4 (*21.7*)

**Table 3:** Intra- and inter-sample Relative Standard Deviations (RSD) of the 4-MBA band area (1550~1615 cm^−1^), and of the adenine band area (700~770 cm^−1^), calculated from SERS maps for Ag substrates excited at 785 nm.

Ag Substrates—785 nm excitation			RSD% Intra Sample (*Inter-Sample*)	
	Average Pore Size	NPs Concentration	w/o Citrate	with Citrate
**4-MBA**	2 µm	1×	38.1, 41.3, 41.2, 36.0 (*6.9*)	29.1, 23.3, 27.9, 31.3 (*26.9*)
		10×	31.3, 24.8, 17.5, 21.1 (*42.6*)	21.5, 20.7, 22.9, 23.3 (*23.4*)
	20 µm	1×	34.5, 32.7, 35.3, 30.1 (*28.4*)	33.4, 29.5, 28.1, 30.1 (*35.6*)
		10×	15.0, 18.2, 19.3 15.0 (*40.0*)	20.0, 29.0, 31.1, 35.3 (*20.2*)
**Adenine**	2 µm	1×	47.3, 27.8, 38.8, 37.8 (*24.7*)	26.4, 24.1, 22.5, 28.0 (*15.2*)
		10×	15.8, 16.9, 12.6, 14.9 (*7.4*)	16.5, 25.0, 19.3, 22.1 (*15.6*)
	20 µm	1×	21.9, 39.0, 21.7, 27.2 (*35.2*)	17.1, 19.6, 19.6, 27.2 (*18.6*)
		10×	22.4, 13.3, 16.3, 14.4 (*16.3*)	33.1, 32.4, 30.0, 34.1 (*14.1*)

## Supplementary Materials

The following are available online at www.mdpi.com/1996-1944/10/12/1365/s1, Figure S1: Transmission Electron Microscopy (TEM) Images of (a) Au and (b) Ag NPs, Figure S2: UV-Visible extinction spectra of Au and Ag NPs; Figure S3: FE-SEM images with the same magnification of Au NPs, as prepared (a,c) or 10× concentrated (b,d), loaded on filter paper in absence (a,b) or presence (c,d) of 20 mM sodium citrate (SC); Figure S4: FE-SEM images with the same magnification of Ag NPs, as prepared (a,c) or 10× concentrated (b,d), loaded on filter paper in absence (a,b) or presence (c,d) of 20 mM sodium citrate (SC); Figure S5: FE-SEM images with the same magnification of Au NPs, as prepared (a,c) or 10× concentrated (b,d), loaded on filter paper in absence (a,b) or presence (c,d) of 20 mM sodium citrate (SC); Figure S6: FE-SEM images with the same magnification of Ag NPs, as prepared (a,c) or 10× concentrated (b,d), loaded on filter paper in absence (a,b) or presence (c,d) of 20 mM sodium citrate (SC); Figures S7–S10: Average SERS spectra of 10 µM Adenine and 4-MBA obtained using different Au and Ag substrates and excitation wavelengths; Figure S11: SERS spectra at several concentrations of 4-MBA on (a) Au substrates, collected at 785 nm and on (b) Ag substrates, collected at 514 nm; Figure S12: Mean and relative standard deviation of the backgrounds of Au solid SERS substrate (in red), acquired at 785 nm, and on Ag solid SERS substrate (in blue), acquired at 514 nm; Figure S13: Raman spectra of 10 µM Adenine and 4-MBA obtained using 514nm and 785 excitation, with the same conditions used for SERS spectra in Figure 3 and Figures S7–S10;Table S1: *R*^2^, LOD and LOQ values calculated from the linear range of the calibration curves of Adenine and 4-MBA on Ag and Au solid SERS substrates.

## Abbreviations

4-MBA	4-Mercaptobenzoic acid
Au	gold
Ag	silver
NPs	nanoparticles
PBS	Phosphate Buffered Saline
RSD	relative standard deviation
LOD	lower limit of detection
LOQ	lower limit of quantification
