# Supplementary material for: Optimization and Characterization of Paper-Made Surface Enhanced Raman Scattering (SERS) Substrates with Au and Ag NPs for Quantitative Analysis
Source: Materials (Basel). 2017 Nov 28;10(12):1365. doi: 10.3390/ma10121365 (PMC5744300; doi:10.3390/ma10121365)
Supplement: Supplementary file 1 [file materials-10-01365-s001.pdf]

Optimization and characterization of paper-made Surface Enhanced Raman Scattering (SERS) substrates with Au and Ag NPs for quantitative analysis

Silvia Dalla Marta[1], Chiara Novara[2], Fabrizio Giorgis[2], Alois Bonifacio[1*] and Valter Sergo[1]

[1] Department of Engineering and Architecture, University of Trieste, Trieste I-34127, Italy
[2] Department of Applied Science and Technology, Politecnico of Torino, Torino I-10129, Italy
[*] Correspondence: abonifacio@units.it; Tel.: +39-040-558-3768

Supplementary Material

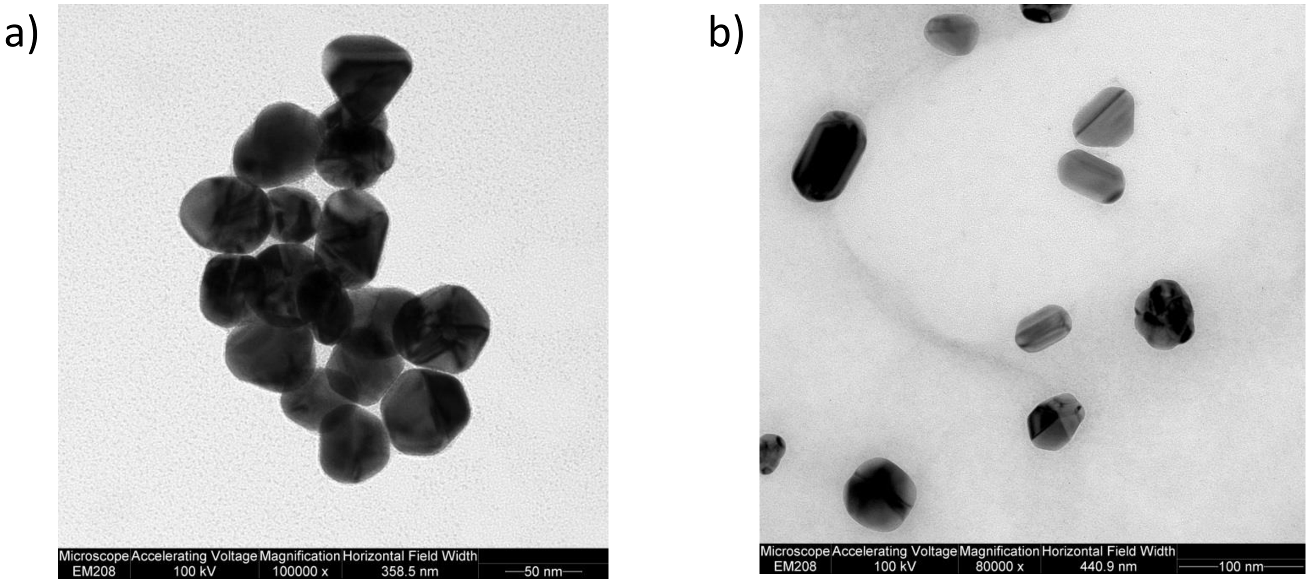

Figure S1: Transmission Electron Microscopy (TEM) Images of a) Au and b) Ag NPs.

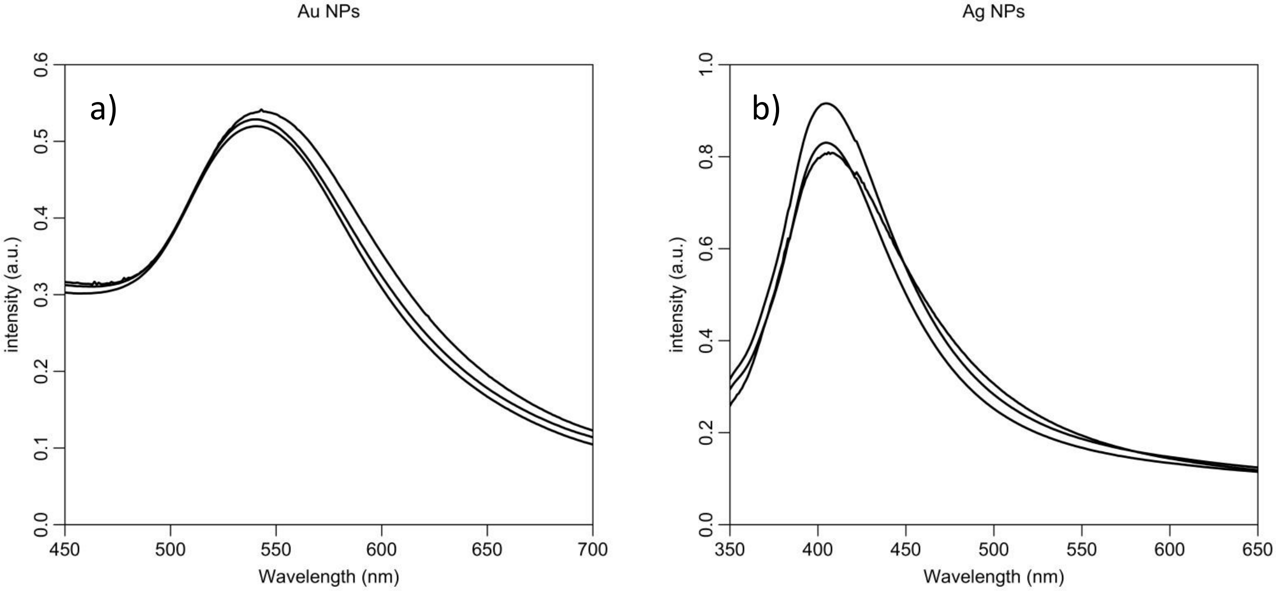

Figure S2: UV-Visible extinction spectra of Au and Ag NPs.

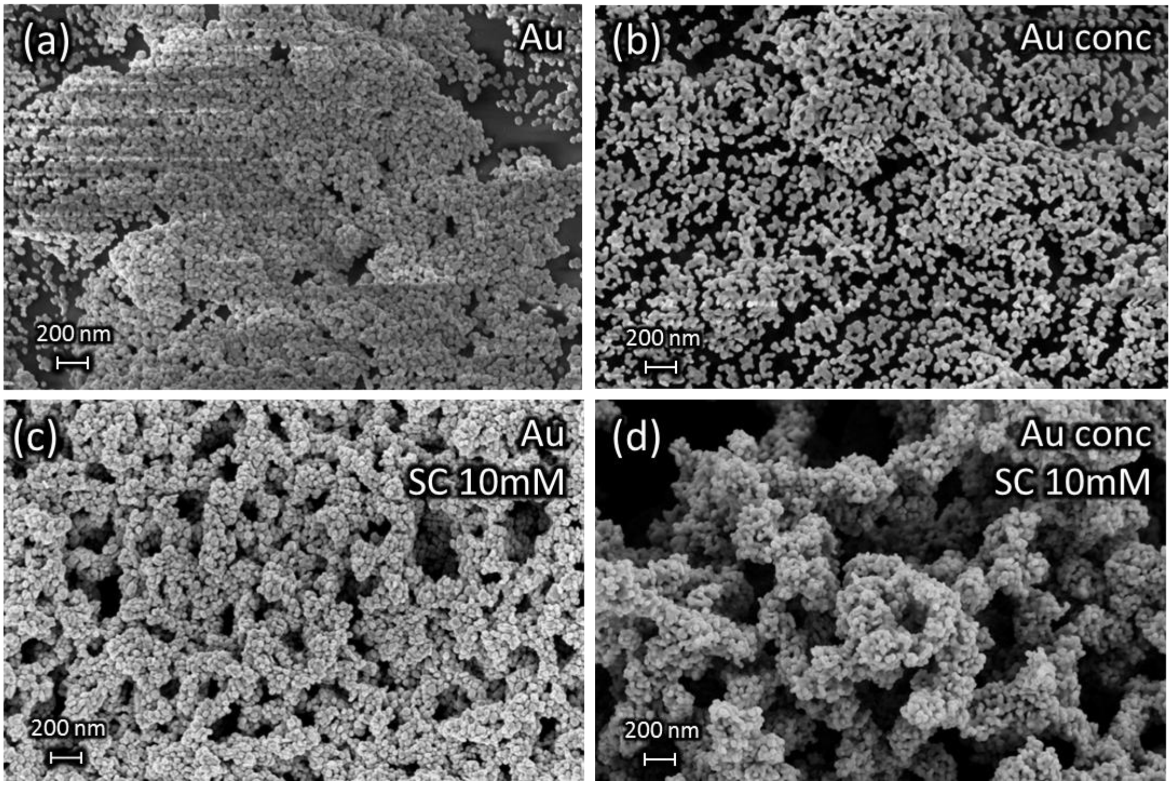

Figure S3: FE-SEM images with the same magnification of Au NPs, as prepared (a, c) or 10x concentrated (b, d), loaded on filter paper (average pore size: 2μm) in absence (a, b) or presence (c, d) of 20 mM sodium citrate (SC).

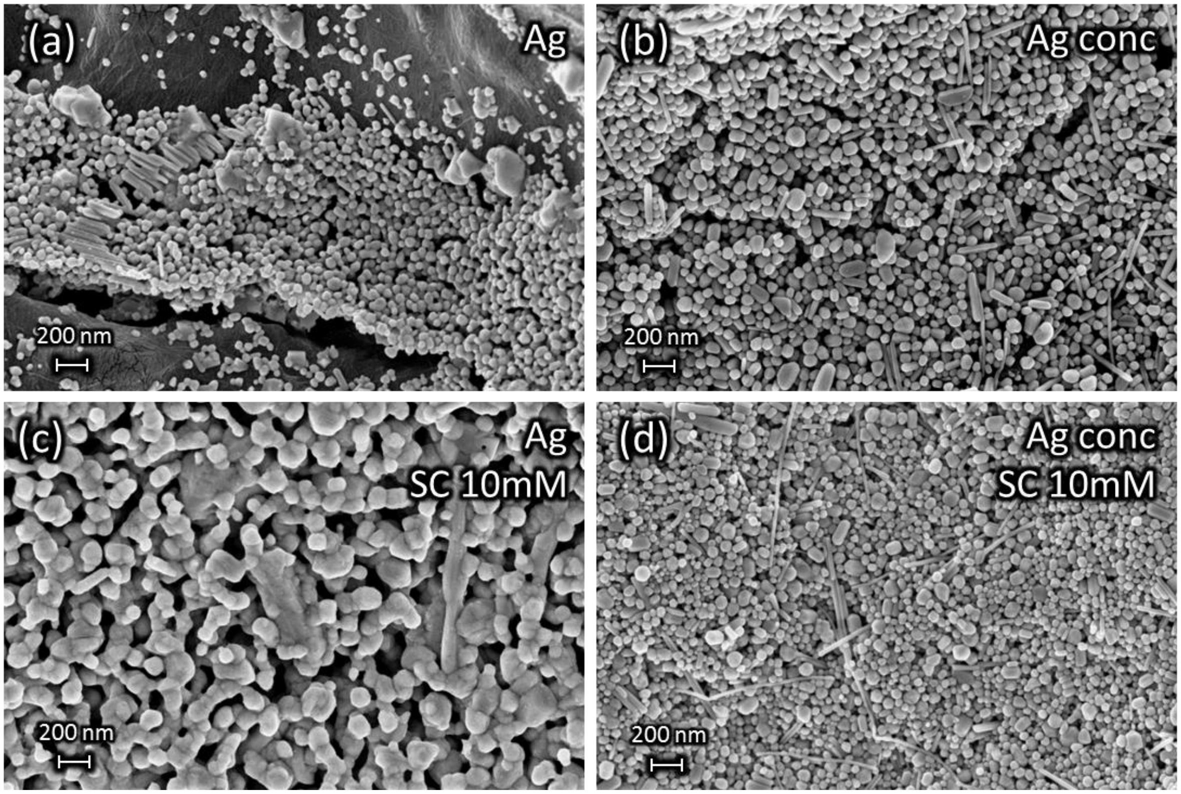

Figure S4: FE-SEM images with the same magnification of Ag NPs, as prepared (a, c) or 10x concentrated (b, d), loaded on filter paper (average pore size: 2μm) in absence (a, b) or presence (c, d) of 20 mM sodium citrate (SC).

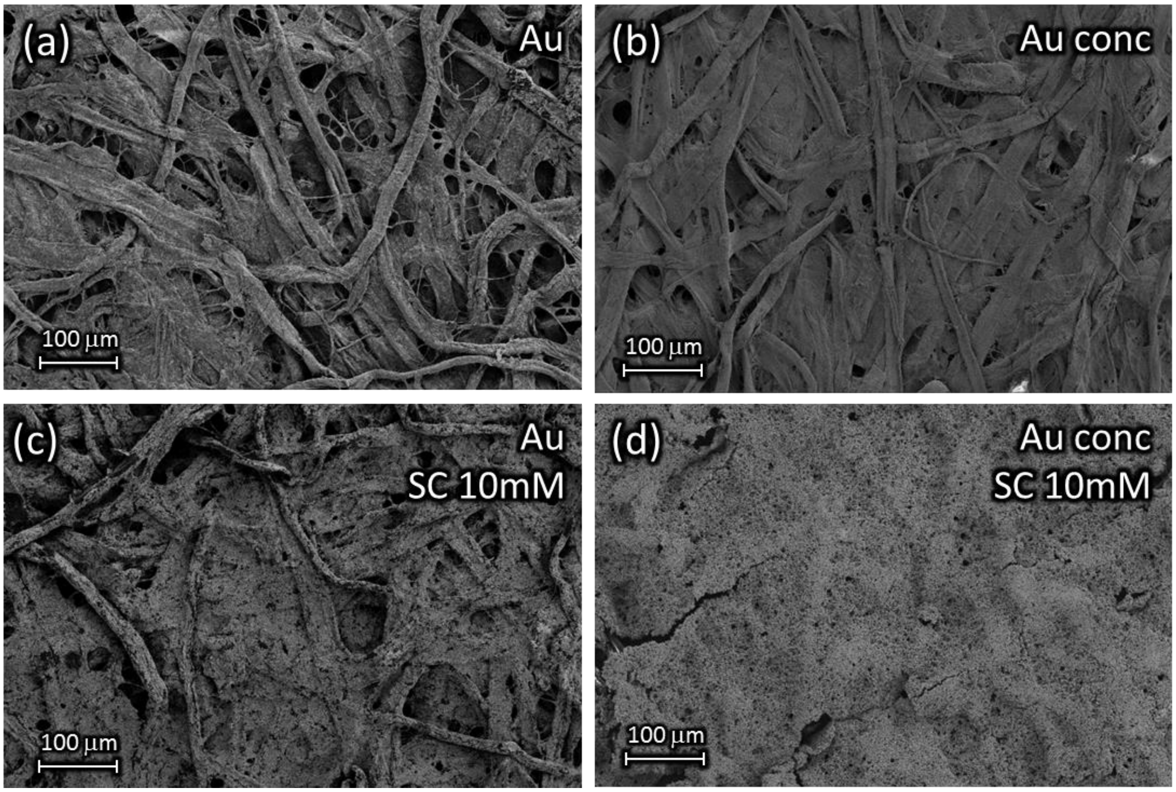

Figure S5: FE-SEM images with the same magnification of Au NPs, as prepared (a, c) or 10x concentrated (b, d), loaded on filter paper (average pore size: 2μm) in absence (a, b) or presence (c, d) of 20 mM sodium citrate (SC).

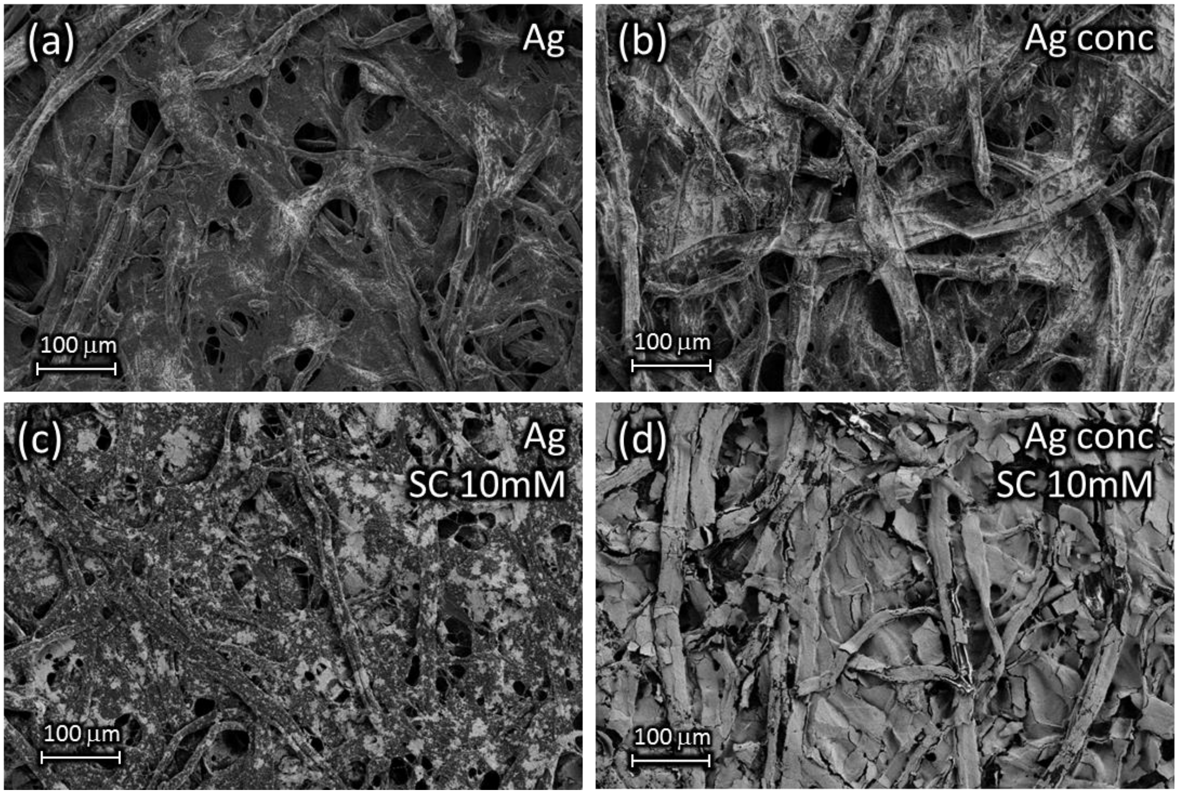

Figure S6: FE-SEM images with the same magnification of Ag NPs, as prepared (a, c) or 10x concentrated (b, d), loaded on filter paper (average pore size: 2μm) in absence (a, b) or presence (c, d) of 20 mM sodium citrate (SC).

Figure S7-S10: Average SERS spectra of 10 μM Adenine and 4-MBA obtained using different Au and Ag substrates and excitation wavelengths. Average intensity and standard deviations (shaded areas representing the interquartile range of the collected data) were calculated over the 64 spectra acquired within each map.

SAMPLE: Adenine – average spectra ± st.dev.				
	**1x 2 µm**	**10x 2 µm**	**1x 20 µm**	**10x 20 µm**
**Au@785**	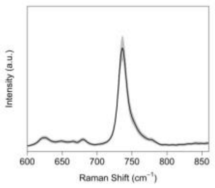	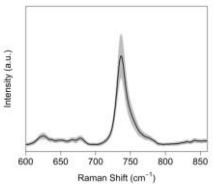	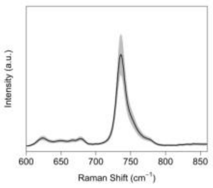	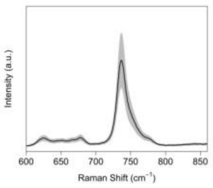
**Ag@514**	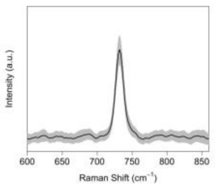	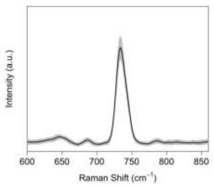	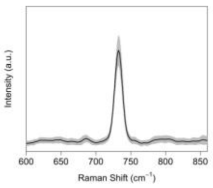	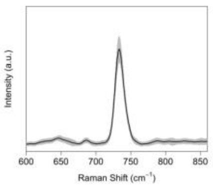
**Ag@785**	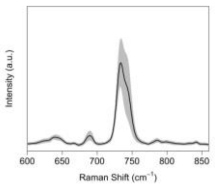	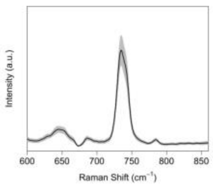	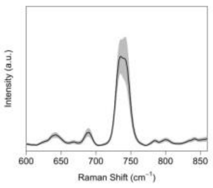	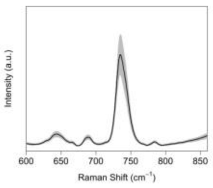

S8

SAMPLE: Adenine – average spectra ± st.dev.				
	**1x 2 µm + Citrate**	**10x 2 µm + Citrate**	**1x 20 µm + Citrate**	**10x 20 µm + Citrate**
**Au@785**	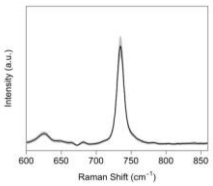	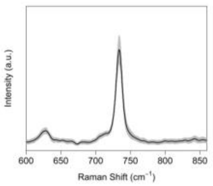	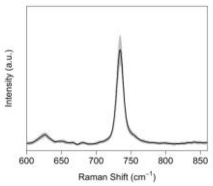	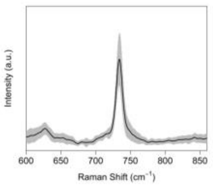
**Ag@514**	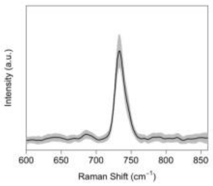	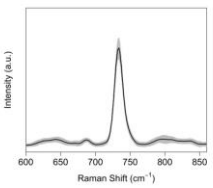	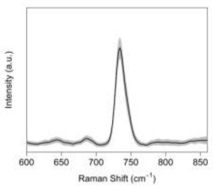	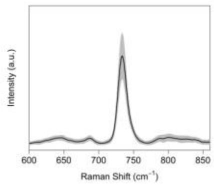
**Ag@785**	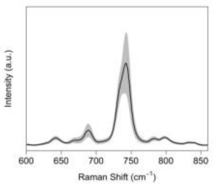	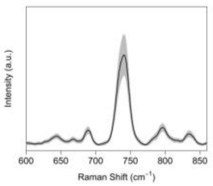	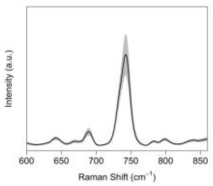	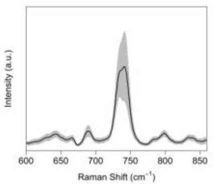

SAMPLE: 4-MBA – average spectra ± st.dev.				
	**1x 2 µm**	**10x 2 µm**	**1x 20 µm**	**10x 20 µm**
**Au@785**	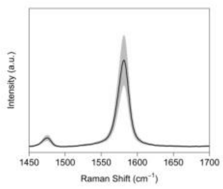	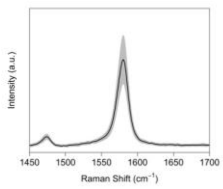	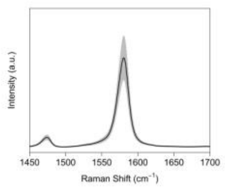	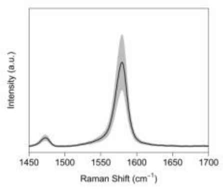
**Ag@514**	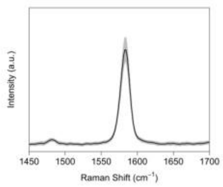	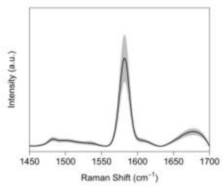	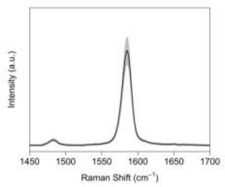	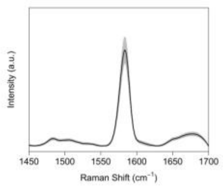
**Ag@785**	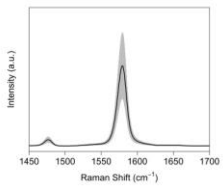	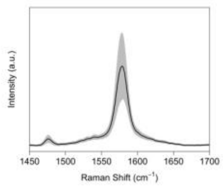	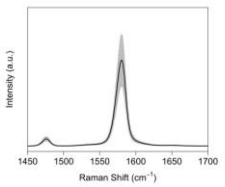	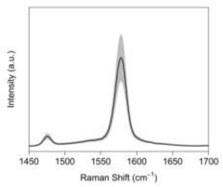

S10

SAMPLE: 4-MBA – average spectra ± st.dev.				
	**1x 2 µm + Citrate**	**10x 2 µm + Citrate**	**1x 20 µm + Citrate**	**10x 20 µm + Citrate**
**Au@785**	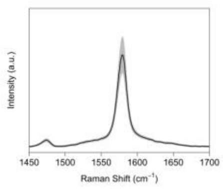	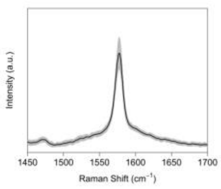	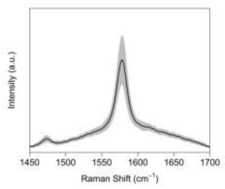	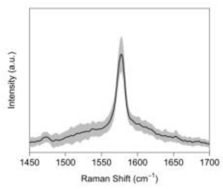
**Ag@514**	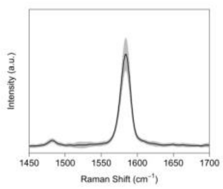	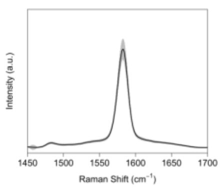	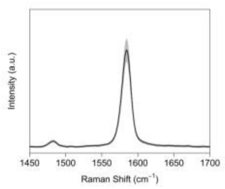	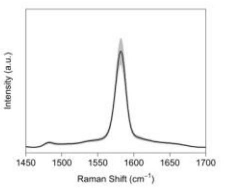
**Ag@785**	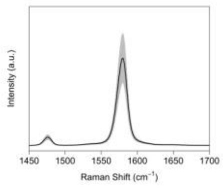	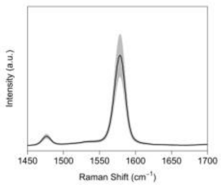	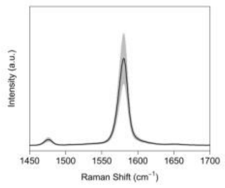	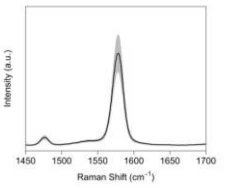

Figure S11: SERS spectra at several concentrations of 4-MBA on a) Au substrates, collected at 785 nm and on b) Ag substrates, collected at 514 nm. Langmuir fitting of the relative calibration curves of b) Adenine on Au and e) on Ag. c) - f)Linear fitting with the

linear range of concentration of the relative calibration curves.

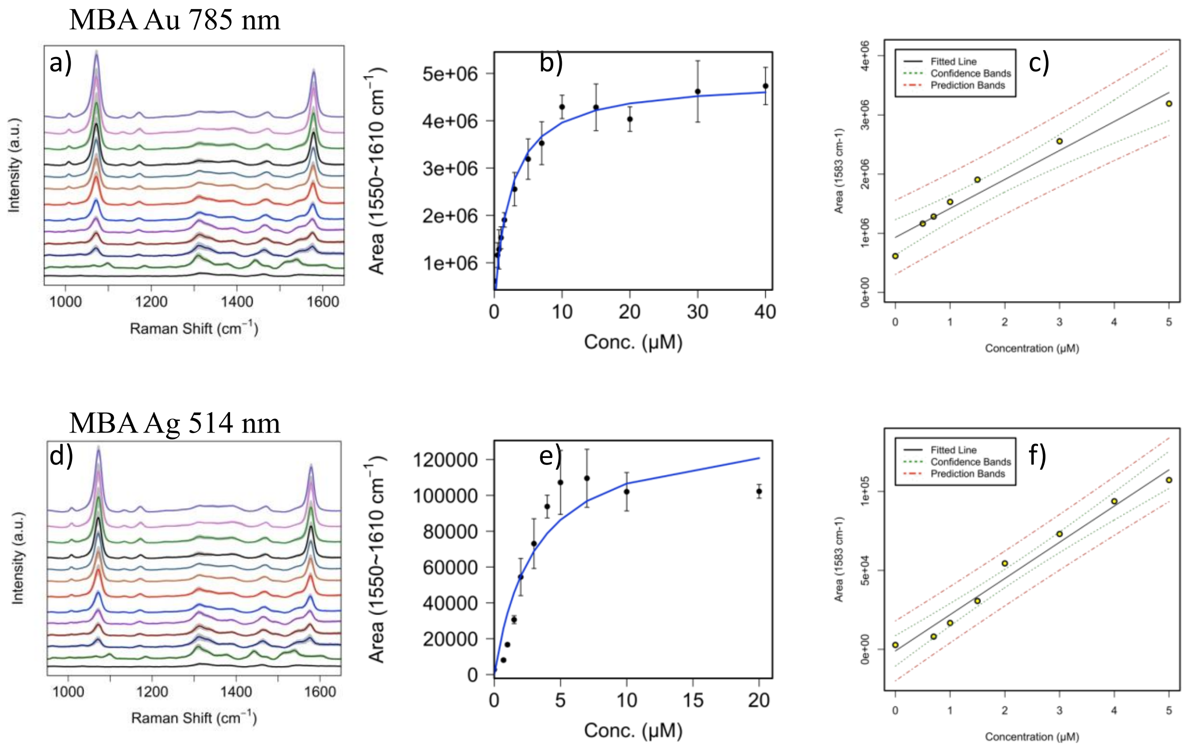

Figure S12: Mean and relative standard deviation of the backgrounds of Au solid SERS substrate (in red), acquired at 785 nm, and on Ag solid SERS substrate (in blue), acquired at 514 nm.

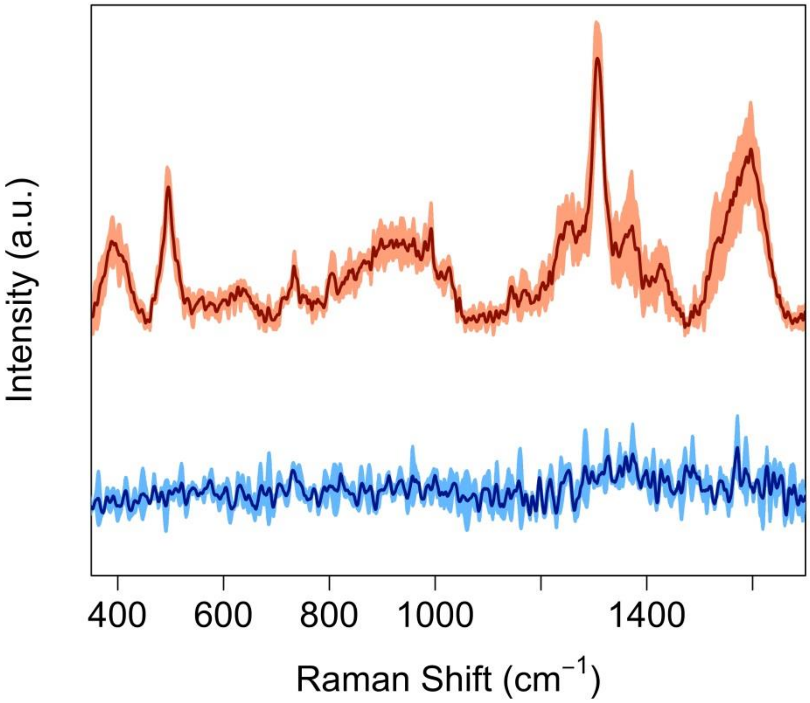

Figure S13: Raman spectra of 10 µM Adenine and 4-MBA obtained using 514nm and 785 excitation, with the same conditions (i.e. same laser power and 5 s exposure time, see Materials and Methods) used for SERS spectra in Figure 3 and Figures S7-S10. At 10 µM, no Raman bands due to adenine or 4-MBA can be observed, only the bands due to bending mode of the water (at ca. 1600 cm^{-1}) and, in the case of the 514 excitation, due to the ν1 and ν4 vibrational modes of the phosphate ion in the buffer (at ca. 1100 and 550 cm^{-1}).

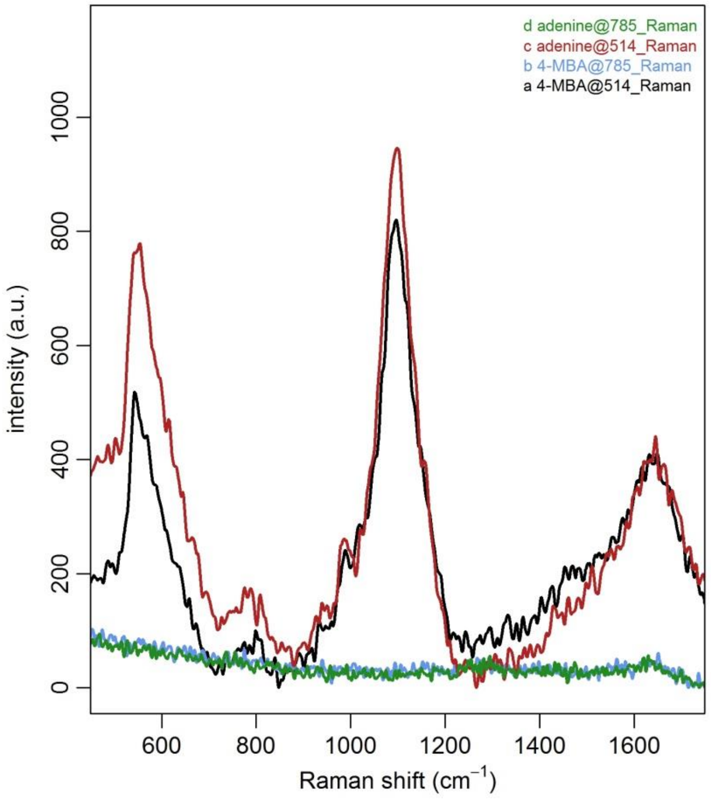

Table S1. R^2, LOD and LOQ values calculated from the linear range of the calibration curves of Adenine and 4-MBA on Ag and Au solid SERS substrates.

		R^2	LOD (µM)	LOQ (µM)
**Au**	**Adenine**	0,9753	0,2928	0,544
	**4-MBA**	0,9408	1,9475	4,071
**Ag**	**Adenine**	0,9585	2,6494	4,835
	**4-MBA**	0,9720	1,2688	2,271
